# Association Between Body Size Phenotypes and Subclinical Atherosclerosis

**DOI:** 10.1210/clinem/dgaa620

**Published:** 2020-09-03

**Authors:** Xavier Rossello, Valentin Fuster, Belén Oliva, Javier Sanz, Leticia A Fernández Friera, Beatriz López-Melgar, José María Mendiguren, Enrique Lara-Pezzi, Héctor Bueno, Antonio Fernández-Ortiz, Borja Ibanez, José María Ordovás

**Affiliations:** 1 Centro Nacional de Investigaciones Cardiovasculares (CNIC), Madrid, Spain; 2 CIBER de Enfermedades CardioVasculares (CIBERCV), Madrid, Spain; 3 Cardiology Department, Health Research Institute of the Balearic Islands (IdISBa), Hospital Universitari Son Espases, Palma, Spain; 4 Icahn School of Medicine at Mount Sinai, New York, New York; 5 Hospital Universitario HM Montepríncipe-CIEC, Madrid, Spain; 6 Universidad CEU San Pablo, Madrid, Spain; 7 Banco de Santander, Madrid, Spain; 8 Hospital Universitario 12 de Octubre and Instituto de Investigación Sanitaria Hospital 12 deOctubre (imas12), Madrid, Spain; 9 Facultad de Medicina, Universidad Complutense de Madrid, Madrid, Spain; 10 Hospital Clínico San Carlos, Universidad Complutense, IdISSC, Madrid, Spain; 11 IIS-Fundación Jiménez Díaz University Hospital, Madrid, Spain; 12 U.S. Department of Agriculture Human Nutrition Research Center on Aging, Tufts University, Boston, Massachusetts; 13 IMDEA Food Institute, CEI UAM+CSIC, Madrid, Spain

**Keywords:** body size phenotypes, obesity, subclinical atherosclerosis, cardiovascular risk

## Abstract

**Context:**

The underlying relationship between body mass index (BMI), cardiometabolic disorders, and subclinical atherosclerosis is poorly understood.

**Objective:**

To evaluate the association between body size phenotypes and subclinical atherosclerosis.

**Design:**

Cross-sectional.

**Setting:**

Cardiovascular disease-free cohort.

**Participants:**

Middle-aged asymptomatic subjects (n = 3909). A total of 6 cardiometabolic body size phenotypes were defined based on the presence of at least 1 cardiometabolic abnormality (blood pressure, fasting blood glucose, triglycerides, low high-density lipoprotein cholesterol, homeostasis model assessment-insulin resistance index, high-sensitivity C-reactive protein) and based on BMI: normal-weight (NW; BMI <25), overweight (OW; BMI = 25.0-29.9) or obese (OB; BMI >30.0).

**Main Outcome Measures:**

Subclinical atherosclerosis was evaluated by 2D vascular ultrasonography and noncontrast cardiac computed tomography.

**Results:**

For metabolically healthy subjects, the presence of subclinical atherosclerosis increased across BMI categories (49.6%, 58.0%, and 67.7% for NW, OW, and OB, respectively), whereas fewer differences were observed for metabolically unhealthy subjects (61.1%, 69.7%, and 70.5%, respectively). When BMI and cardiometabolic abnormalities were assessed separately, the association of body size phenotypes with the extent of subclinical atherosclerosis was mostly driven by the coexistence of cardiometabolic risk factors: adjusted OR = 1.04 (95% confidence interval [CI], 0.90-1.19) for OW and OR = 1.07 (95% CI, 0.88-1.30) for OB in comparison with NW, whereas there was an increasing association between the extent of subclinical atherosclerosis and the number of cardiometabolic abnormalities: adjusted OR = 1.21 (95% CI, 1.05-1.40), 1.60 (95% CI, 1.33-1.93), 1.92 (95% CI, 1.48-2.50), and 2.27 (95% CI, 1.67-3.09) for 1, 2, 3, and >3, respectively, in comparison with noncardiometabolic abnormalities.

**Conclusions:**

The prevalence of subclinical atherosclerosis varies across body size phenotypes. Pharmacologic and lifestyle interventions might modify their cardiovascular risk by facilitating the transition from one phenotype to another.

Obesity is an expanding worldwide public health epidemic with enormous medical and socioeconomic consequences (1). Obesity causes a decline in life expectancy due to its association with metabolic and cardiovascular disorders (1, 2). Nevertheless, the underlying relationship between weight, cardiometabolic disorders, and cardiovascular risk is complex and far from being understood. On the one hand, it is widely recognized that obesity-related cardiometabolic disturbances, such as dyslipidemia, glucose intolerance, or hypertension, are heterogeneously distributed across individuals with similar body mass index (BMI). This has resulted in the identification of body size phenotypes: subsets of individuals either apparently protected or prone to the development of cardiometabolic abnormalities associated with overweight and obesity (ie, metabolically healthy obesity (3)). On the other hand, some controversial data suggest that metabolically healthy obese subjects may not be at increased risk for cardiovascular events in comparison to their nonobese peers (4). In this context of poor understanding of the impact of body size phenotypes on cardiovascular risk, there is a need to comprehensively evaluate the distribution of subclinical atherosclerosis across body size phenotypes.

There is currently no universal definition of body size phenotypes based on obesity measures and cardiometabolic abnormalities. Obesity is mostly defined using BMI, although waist circumference (WC) criteria are sometimes used instead. More problems are found when determining a metabolically healthy status. Generally, it has been defined based on the components of the metabolic syndrome with the addition of an insulin resistance index and serum inflammatory markers (3, 5). This definition is of utmost importance because metabolic health has been more closely associated with subclinical atherosclerosis than obesity (5-7).

Using a large cohort of asymptomatic middle-aged subjects without known cardiovascular disease, this study aimed to: (a) determine the prevalence of each of the 6 body size phenotypes (normal-weight, overweight, and obese; with or without cardiometabolic abnormalities) as well as their defining components (BMI and cardiometabolic status) separately; (b) evaluate both the presence and extent of subclinical atherosclerosis by body size phenotypes; and (c) assess the distribution of traditional risk factors, lifestyle factors, and psychosocial characteristics among body size phenotypes and to obtain adjusted estimates evaluating the association between body size phenotypes and subclinical atherosclerosis.

## Materials and Methods

### Study overview

The Progression of Early Subclinical Atherosclerosis (PESA) study is an observational cohort of 4184 subjects, which aims to understand the determinants of the onset and progression of subclinical atherosclerosis diagnosed by noninvasive vascular imaging in multiple vascular sites in a middle-aged population recruited among employees of the Bank Santander Headquarters in Madrid, Spain. Further details of the study design and data collection have been reported elsewhere (8). The study protocol was approved by the Ethics Committee of Instituto de Salud Carlos III (Madrid, Spain). All participants provided written informed consent (8).

### Study participants

Volunteers from 40 to 54 years old were prospectively included in this cohort if at baseline they were free of any cardiovascular or chronic kidney disease, were not under active treatment for cancer, did not have previous transplantation, did not exceed a BMI of 40 kg/m^2^, and did not have any disease that might jeopardize life expectancy by the end of the expected follow-up period (6 years). Among the initial participants, 275 (6.6%) were excluded from this analysis (262 because of incomplete imaging studies and 13 because of a lack of information regarding cardiometabolic abnormalities). The final sample consisted, therefore, of 3909 participants.

### Measurement of body mass index and cardiometabolic components

**Figure 1.**
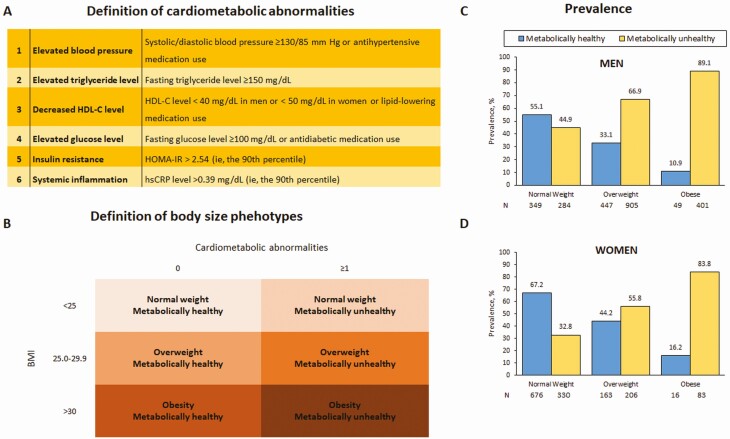
Definition of cardiometabolic abnormalities and body size phenotypes and description of their prevalence by gender. Abbreviations: BMI, body mass index (kg/m^2^); HDL-C, high-density lipoprotein–cholesterol; HOMA-IR, homeostasis model assessment of insulin resistance; hsCRP, high-sensitivity C-reactive protein. HOMA was calculated through the following formula: Fasting Serum Insulin Level (Microunits per Milliliter) × Fasting Plasma Glucose Level (Millimoles per Liter)/22.5.

Anthropometric data (height, weight, and WC) were collected as previously reported and through standardized procedures according to the PESA study protocol (8-12). The 6 cardiometabolic components were defined according to the criteria most commonly reported in the literature (3, 13, 14) (Fig. 1): (a) blood pressure ≥130/85 mm Hg or antihypertensive medication use; (b) fasting triglyceride level ≥150 mg/dL; (c) high-density lipoprotein–cholesterol (HDL-C) level <40 mg/dL in men or <50 mg/dL in women or lipid-lowering medication use; (d) fasting glucose level ≥100 mg/dL or antidiabetic medication use; (e) homeostasis model assessment of insulin resistance (HOMA-IR) >2.45 (the 90th percentile); and (f) high-sensitivity C-reactive protein (hsCRP) level >0.39 mg/L (the 90th percentile) (3, 15). In addition to hsCRP, other systemic markers are also reported (16), though they were not used to define cardiometabolic abnormalities.

### Body size phenotype definitions

Based on their BMI, individuals were classified as being normal-weight (BMI <25 kg/m^2^), overweight (BMI, 25.0-29.9 kg/m^2^), or obese (BMI > 30.0 kg/m^2^). The presence of 1 or more of the 6 cardiometabolic components was defined as “metabolically unhealthy,” whereas the lack of all these components was defined as “metabolically healthy.”

For each participant, body size phenotype was defined based on the combination of the BMI category (normal, overweight, or obese) and the cardiometabolic category (healthy or unhealthy), resulting in 6 categories (Fig. 1).

### Assessment of subclinical atherosclerosis

The primary outcomes were obtained using two-dimensional vascular ultrasound (2DVUS) and noncontrast cardiac computed tomography (CCT) in all participants, as previously described (9). Briefly, the presence of atherosclerotic plaques by ultrasound was assessed by a cross-sectional sweep of carotids, infrarenal abdominal aorta, and iliofemoral arteries. Plaques were defined as focal protrusions into the arterial lumen of thickness >0.5 mm or >50% of the surrounding intima-media thickness, or as a diffuse intima-media thickness >1.5 mm (17). The Agatston method was used to estimate the coronary artery calcium score (CACS) from CCT images (18).

Both measures, the presence of atherosclerotic plaques by vascular ultrasound and a CACS value ≥1, were combined to define the extent of subclinical atherosclerosis. Multiterritorial extent was defined according to the number of vascular sites with evidence of disease, including right carotid, left carotid, abdominal aorta, right iliofemoral, left iliofemoral, and coronary arteries. Participants were classified as disease-free (0 vascular sites affected) or having focal (1 site), intermediate (2 to 3 sites), or generalized atherosclerosis (4 to 6 sites) (10).

All images were analyzed at a central Imaging Core Laboratory by experienced operators who were blinded to participant data.

### Traditional risk predictors, lifestyle factors, and psychosocial characteristics

Potential confounders, such as traditional risk factors (age, gender, and smoking status; all present in main risk scores) (19), lifestyle factors (physical activity level, alcohol intake, resting sleeping patterns, and eating patterns) and psychosocial characteristics (stress and depression) were used for adjustment to assess the association between subclinical atherosclerosis and body size phenotypes. Physical activity level and resting sleeping patterns were evaluated using actigraphy-measured data (12). The quantity of sleep was assessed by triaxial accelerometry, using Acti Trainers accelerometers (Actigraph, Pensacola, Florida) placed on the participant’s waist for 7 days (12). Eating patterns were evaluated according to a previous cluster definition based on dietary habits obtained in this cohort (Mediterranean, Western, and Social-Business) (12). Depression and perceived stress at home and work were evaluated using the CES-D (Center for Epidemiological Studies Depression) (20) and PSS (Perceived Stress Scale) questionnaires (21), respectively. All interviews were conducted blinded to clinical, laboratory, and imaging results by trained technicians.

Given that some lipid disorders were already included in the definition of body size phenotypes, the addition of low-density lipoprotein–cholesterol (LDL-C) levels to the model might over adjust the model. Nevertheless, considering the importance of LDL-C (9), we also performed an additional sensitivity analysis adjusting for this risk factor.

### Statistical analysis

Categorical variables are described as frequency (%) and compared between groups using chi-square tests, while continuous baseline data were expressed as mean ± standard deviation, or median (interquartile range), and differences were accordingly compared using the *t* test with or without log transformation for normal and nonnormal distributed data, respectively. Univariate and multivariate regression models were used to assess the evaluation between the study variables (BMI, cardiometabolic abnormalities, or body size phenotypes) and the main outcomes: (a) ordinal models for number of atherosclerotic plaques and multi-territorial extent; and (b) logistic models for CACS (0 vs ≥1). To control the potential measured confounding from the association between body size phenotypes and the outcomes related to subclinical atherosclerosis, estimates were adjusted for age, gender, and smoking status, physical activity level, alcohol intake, resting sleeping patterns, eating patterns, and psychosocial characteristics (CES-D and PSS). Additionally, we also adjusted for LDL-cholesterol in a sensitivity analysis. All analyses were performed using STATA software version 15.1 (Stata Corp, College Station, TX, USA). GraphPad Prism version 6.00 (GraphPad Software, La Jolla California, USA) was used to perform some graphs.

## Results

### Prevalence and characteristics of body size phenotypes

Compared with women (n = 1474; 37.7%), men were more often metabolically unhealthy (65.3% vs 42.0%; *P* < 0.001) and had lesser prevalence of normal-weight (26.0% vs 68.3%; *P* < 0.001). The by-gender distribution of the body size phenotypes is shown in Fig. 1C and 1D. Within each stratum of BMI, men had a higher percentage of metabolically unhealthy subjects than women. Metabolically unhealthy status was predominant in overweight and obese individuals regardless of their gender. The most common phenotype for men was overweight/metabolically unhealthy, whereas women were predominantly normal-weight/metabolically healthy.

Demographic factors and clinical characteristics related to cardiometabolic abnormalities are described by body size phenotype in Table 1. Within each BMI strata, unhealthy individuals had a higher prevalence of family history of cardiovascular disease compared with metabolically healthy subjects. Similarly, mean WC was consistently higher in metabolically unhealthy subjects within each BMI strata.

**Table 1.** Clinical Characteristics of the Study Population (n = 3909) by Body Size Phenotypes

	Overall	Normal-weight		Overweight		Obese	
		Metabolically healthy	Metabolically unhealthy	Metabolically healthy	Metabolically unhealthy	Metabolically healthy	Metabolically unhealthy
**Baseline characteristics**							
Prevalence, n (%)	3909 (100%)	1025 (26.2%)	614 (15.7%)	610 (15.6%)	1111 (28.4%)	65 (1.7%)	484 (12.4%)
Age (years), mean ± SD	45.7 ± 4.2	44.5 ± 3.8	45.3 ± 4.2	45.5 ± 4.2	46.5 ± 4.3	46.5 ± 4.0	47.0 ± 4.3^ns^
Men, n (%)	2435 (62.3)	349 (34.0)	284 (46.3)	447 (73.3)	905 (81.5)	49 (75.4)	401 (82.9)^ns^
Family history of CVD, n (%)	610 (15.6)	122 (11.9)	104 (16.9)	84 (13.8)	196 (17.6)	6 (9.2)	98 (20.2)
CVRF							
BMI (kg/m^2^), mean ± SD	26.1 ± 3.8	22.4 ± 1.7	23.0 ± 1.6	26.9 ± 1.3	27.4 ± 1.4	31.5 ± 1.5	32.8 ± 2.2
Weight (kg), mean ± SD	76.3 ± 14.7	63.2 ± 8.8	65.9 ± 9.1	79.7 ± 8.8	82.0 ± 8.8	92.3 ± 9.0	97.7 ± 10.7
Waist circumference (cm), mean ± SD	89.0 ± 11.9	77.8 ± 7.0	81.1 ± 7.2	91.2 ± 6.8	94.1 ± 6.5	101.1 ± 6.7	106.9 ± 7.7
Central obesity, n (%)	706 (18.1)	6 (0.6)	6 (1.0)^ns^	69 (11.3)	198 (17.8)	36 (55.4)	391 (80.8)
Hypertension, n (%)	438 (11.2)	0 (0.0)	64 (10.4)	0 (0.0)	222 (20.0)	0 (0.0)	152 (31.4)
SBP (mm Hg), mean ± SD	116.0 ± 12.5	108.3 ± 9.1	113.3 ± 12.7	114.4 ± 7.9	121.4 ± 12.2	116.5 ± 8.0	125.7 ± 12.7
DBP (mm Hg), mean ± SD	72.3 ± 9.4	66.6 ± 6.8	70.4 ± 9.1	70.9 ± 6.6	75.9 ± 9.3	73.3 ± 6.0	80.3 ± 9.0
Antihypertensive therapy, n(%)	280 (7.2)	0 (0.0)	42 (6.8)	0 (0.0)	146 (13.1)	0 (0.0)	92 (19.0)
Dyslipidemia, n (%)	1604 (41.0)	103 (10.0)	275 (44.8)	140 (23.0)	755 (68.0)	17 (26.2)	314 (64.9)
Lipid-lowering therapy, n(%)	264 (6.8)	0 (0.0)	57 (9.3)	0 (0.0)	143 (12.9)	0 (0.0)	64 (13.2)
Total cholesterol (mg/dL), mean ± SD	200.3 ± 33.1	195.3 ± 29.1	189.7 ± 31.3	206.0 ± 31.8	205.1 ± 34.9^ns^	206.0 ± 32.1	205.7 ± 36.1^ns^
LDL-C (mg/dL), mean ± SD	132.2 ± 29.5	122.5 ± 26.3	126.1 ± 27.0	137.5 ± 28.7	139.3 ± 30.6^ns^	138.7 ± 30.4	136.8 ± 30.2^ns^
HDL-C (mg/dL), mean ± SD	49.2 ± 12.3	59.5 ± 10.9	46.5 ± 10.7	52.7 ± 9.8	42.6 ± 9.6	50.3 ± 8.3	41.3 ± 8.4
Triglycerides (mg/dL), median [IQR]	79.0 [59.0–112.0]	61.0 [50.0-77.0]	71.0 [56.0-98.0]	74.0 [58.0-96.0]	102.0 [75.0-139.0]	78.0 [64.0-98.0]	115.5 [86.0-159.0]
Diabetes mellitus, n (%)	69 (1.8)	0 (0.0)	8 (1.3)	0 (0.0)	28 (2.5)	0 (0.0)	33 (6.8)
Antidiabetic therapy, n (%)	52 (1.3)	0 (0.0)	7 (1.1)	0 (0.0)	25 (2.3)	0 (0.0)	20 (4.1)^ns^
Fasting glucose (mg/dL), median (IQR)	89.0 [83.0-95.0]	85.0 [80.0-89.0]	86.0 [82.0-93.0]	88.0 [83.0-93.0]	93.0 [87.0-99.0]	90.0 [86.0-95.0]	96.0 [90.0-102.5]
Hemoglobin A_1c_. %, median [IQR]	5.4 [5.2-5.6]	5.3 [5.1-5.5]	5.4 [5.2-5.6]	5.4 [5.1-5.6]	5.5 [5.2-5.7]	5.5 [5.1-5.6]	5.5 [5.3-5.8]
HOMA-IR (mg/dL), median [IQR]	1.1 [0.8-1.7]	0.8 [0.6-1.0]	0.9 [0.7-1.3]	1.0 [0.7-1.4]	1.5 [1.1-2.1]	1.5 [1.1-1.9]	2.3 [1.6-3.1]
hsCRP (mg/dL), median [IQR]	0.10 [0.05-0.19]	0.06 [0.03-0.10]	0.08 [0.04-0.17]	0.09 [0.05-0.16]	0.12 [0.07-0.23]	0.13 [0.08-0.19]	0.21 [0.12-0.40]
High erythrocyte sedimentation rate (1h)	23 (0.6)	4 (0.4)	5 (0.8)^ns^	1 (0.2)	10 (0.9)^ns^	1 (1.5)	2 (0.4)^ns^
Ferritin (ng/mL)	57.8 [25.7-133.4]	34.4 [16.0-64.7]	46.3 [20.6-89.5]	76.5 [33.8-143.9]	108.9 [50.9-191.1]	70.0 [25.9-135.2]	123.3 [52.5-210.7]

Erythrocyte sedimentation rate was available for 3908, whereas ferritin was available for 2342 participants. The remaining baseline characteristics were available for all subjects. High erythrocyte sedimentation rate was defined by the most widely equation, which applies differently to men and women (16).

Abbreviations: BMI, body mass index; CVD, cardiovascular disease; DBP, diastolic blood pressure; HDL-C, high-density lipoprotein–cholesterol; HOMA-IR, homeostasis model assessment of insulin resistance; hsCRP, high-sensitivity C-reactive protein; IQR, interquartile range; LDL-C, low-density lipoprotein–cholesterol; SBP, systolic blood pressure.

^ns^nonsignificant for the comparison between healthy and unhealthy within each BMI strata. The rest of comparison were statistically significant.

### Outcomes

**Figure 2.**
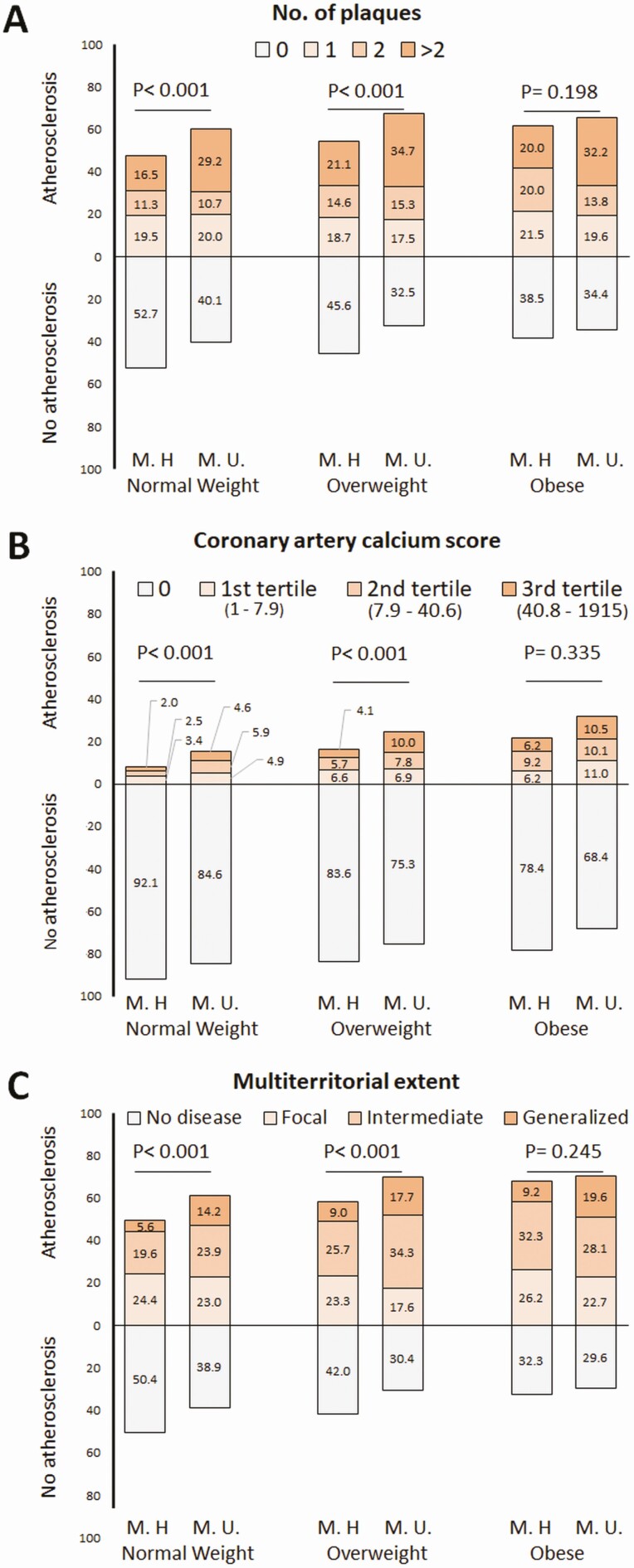
Subclinical atherosclerosis assessed by 2D-vascular ultrasound and noncontrast cardiac computed tomography (CCT) in all participants (observed outcomes). Percentages of outcomes within each body size phenotype. Number of plaques (panel A) was assessed using two-dimensional vascular ultrasound, whereas coronary artery calcium score (panel B) was obtained by noncontrast CCT. The multi-territorial extent (panel C) was defined by the combination of both imaging techniques and classified subjects as disease-free (0 vascular sites affected) or having focal (1 site), intermediate (2 to 3 sites), or generalized atherosclerosis (4 to 6 sites) (10). Abbreviations: M.H., metabolically healthy; M.U., metabolically unhealthy.

Outcomes (subclinical atherosclerosis assessed by 2DVUS and CACS) are described in Fig. 2. Among nonobese subjects (normal-weight and overweight), unhealthy individuals had higher prevalence of subclinical atherosclerosis and multi-territorial extent compared with metabolically healthy subjects. In contrast, both healthy and unhealthy obese subjects had no significant differences in these outcomes, except for a higher number of territories with plaques in the unhealthy subgroup. The extension of subclinical atherosclerosis (Fig. 2C) steadily increased across BMI categories among healthy subjects, whereas for unhealthy subjects, the extension of the disease was similarly high for overweight and obese when compared with normal-weight individuals.

### Adjusted association between subclinical atherosclerosis and body size phenotypes

To control the potential measured confounding from the association between subclinical atherosclerosis and body size phenotypes, estimates were adjusted for age, gender, smoking status, physical activity level, alcohol intake, resting sleeping patterns, eating patterns, and psychosocial characteristics. The distribution of these demographic data, lifestyle factors, and psychosocial characteristics by body size phenotypes is reported in Tables 1 and 2.

**Table 2.** Lifestyle Factors and Psychosocial Characteristics by Body Size Phenotypes

	Overall	Normal-weight		Overweight		Obese	
		Metabolically healthy	Metabolically unhealthy	Metabolically healthy	Metabolically unhealthy	Metabolically healthy	Metabolically unhealthy
**Smoking status**							
*Current smoking, n (%)*	798 (20.6)	176 (17.4)	170 (27.8)	92 (15.3)	261 (23.8)	9 (13.8)	90 (18.7)^ns^
**Physical activity level**							
*Moderate/Vigorous (min/day)*, median [IQR]	34.7 [22.8-49.8]	34.3 [22.5-48.8]	33.8 [22.4-50.3]^ns^	38.0 [24.7-51.7]	36.7 [23.8-50.7]^ns^	33.2 [24.0-50.0]	31.3 [20.5-45.2]^ns^
**Alcohol intake**							
*Ethanol gr/day*, median [IQR]	6.4 [1.6-14.5]	4.7 [1.0-10.6]	5.0 [0.9-11.1]^ns^	7.9 [2.8-15.9]	7.7 [2.1-16.6]^ns^	12.1 [5.1-25.5]	8.2 [1.9-20.9]^ns^
**Resting sleeping patterns**							
*<6h, n (%)*	1030 (26.5)	211 (20.7)	127 (20.8)^ns^	149 (24.5)	333 (30.1)	27 (41.5)	183 (37.9)^ns^
*6-7h, n (%)*	1498 (38.5)	367 (36.0)	234 (38.3)^ns^	255 (42.0)	443 (40.1)^ns^	22 (33.9)	177 (36.6)^ns^
*≥7h, n (%)*	1364 (35.0)	442 (43.3)	250 (40.9)^ns^	203 (33.5)	330 (29.8)^ns^	16 (24.6)	123 (25.5)^ns^
**Eating Pattern**							
*Mediterranean, n (%)*	1541 (40.3)	511 (51.3)	266 (44.5)	223 (37.4)	372 (33.9)^ns^	26 (40.6)	143 (30.4)^ns^
*Western, n (%)*	1576 (41.2)	386 (38.8)	260 (43.5)^ns^	245 (41.1)	485 (44.2)^ns^	19 (29.7)	181 (38.4)^ns^
*Social-Business, n (%)*	705 (18.4)	99 (9.9)	72 (12.0)^ns^	128 (21.5)	240 (21.9)^ns^	19 (29.7)	147 (31.2)^ns^
**Psychosocial characteristics**							
*CES-D score*, median [IQR]	4.0 [2.0-8.0]	4.0 [2.0-9.0]	4.0 [2.0-8.0]^ns^	4.0 [1.0-8.0]	4.0 [2.0-8.0]^ns^	4.0 [2.0-7.0]	4.0 [2.0-8.0]^ns^
*CES-D score ≥ 16 (being depressed), n (%)*	245 (6.3)	69 (6.7)	37 (6.0)^ns^	29 (4.8)	66 (5.9)^ns^	5 (7.7)	39 (8.1)^ns^
*PSS*, median [IQR]	17.0 [13.0-22.0]	18.0 [13.0-22.0]	17.0 [13.0-22.0]^ns^	17.0 [12.0-22.0]	16.0 [12.0-21.0]^ns^	17.0 [12.0-22.0]	17.0 [13.0-22.0]^ns^
*PSS score ≥ 25 (being stressed), n (%)*	559 (14.3)	134 (13.1)	77 (12.6)^ns^	90 (14.8)	152 (13.7)^ns^	11 (16.9)	95 (19.6)^ns^

The percentage of missing data was 1.0% (n = 3870) for smoking status; 1.2% (n = 3861) for physical activity level; 0.1% (n = 3906) for alcohol intake; 0.4% (n = 3892) for resting sleeping patterns; 2.2% for eating patterns; and 0.03 (n = 3908) for psychosocial characteristics.

Depression and perceived stress at home and at work were evaluated using CES-D (Center for Epidemiological Studies Depression) and PSS (Perceived Stress Scale) questionnaires.

Abbreviations: CES-D, Center for Epidemiological Studies Depression; IQR, interquartile range; PSS, Perceived Stress Scale.

^ns^nonsignificant for the comparison between healthy and unhealthy within each BMI strata. The rest of comparison were statistically significant.

**Figure 3.**
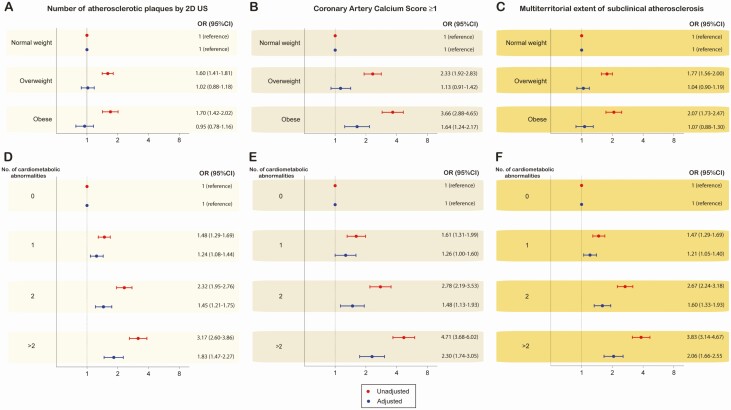
Unadjusted and adjusted risk for subclinical atherosclerosis by body mass index and cardiometabolic abnormalities. Unadjusted (red estimates) and adjusted (blue estimates) are both reported. Multivariate models were adjusted for age, gender, smoking status, physical activity level, alcohol intake, resting sleeping patterns, eating patterns, and psychosocial characteristics.

In the first step, BMI and cardiometabolic abnormalities were studied separately to further dissect the association between subclinical atherosclerosis and body size phenotypes after adjustment for confounding (Fig. 3). Whereas the number of plaques, positive CACS, and multiterritorial extent increased alongside the increase in the number of cardiometabolic abnormalities, there was no increase of subclinical disease across BMI categories for the number of atherosclerotic plaques and the extent of the disease and a weak positive trend for positive CACS.

**Figure 4.**
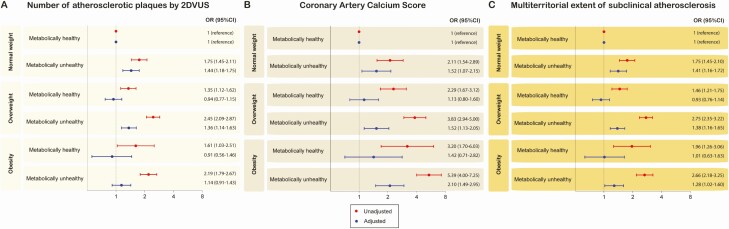
Unadjusted and adjusted associations between subclinical atherosclerosis and body size phenotypes. Panel A shows OR (95% CI) for the number of plaques assessed by two-dimensional vascular ultrasound (2DVUS), whereas panel B displays the estimates for coronary artery calcium score, and panel C for the multi-territorial extent. Unadjusted (black estimates) and adjusted (blue estimates) are reported in all panels. The model was adjusted by age, gender, smoking status, physical activity level, alcohol intake, resting sleeping patterns, eating patterns, and psychosocial characteristics.

In the second step, we evaluated the association between subclinical atherosclerosis and body size phenotypes after adjusting for confounding factors (Fig. 4). Among metabolically healthy subjects, overweight and obesity were not associated with an increased risk for subclinical disease. Among metabolically unhealthy subjects, overweight and obesity were generally associated with a higher risk for subclinical atherosclerosis, except for the number of atherosclerotic plaques in obese-unhealthy subjects.

### Sensitivity analyses

Even though mean LDL-C did not significantly differ between the metabolically healthy and the unhealthy overweight and obese groups (Table 1), we replicated the main findings adjusting for LDL-C on top of the other potential confounding factors without obtaining substantial differences in the association between body size phenotypes and subclinical atherosclerosis (data not shown).

## Discussion

### Principal findings

Our main findings can be summarized as follows: (a) the most common phenotype for middle-aged men was overweight/metabolically unhealthy, whereas women were predominantly normal-weight/metabolically healthy; (b) for metabolically healthy subjects, the presence of subclinical atherosclerosis increases across BMI categories, whereas for metabolically unhealthy subjects, fewer differences were observed between overweight and obese subjects in terms of 2DVUS and CACS findings in comparison with their normal-weight counterparts; and (c) the association of BMI with subclinical atherosclerosis seems to be mostly driven by the coexistence of cardiometabolic risk factors.

### Implications of using different body size phenotypes definitions

The prevalence and characteristics of body size phenotypes are highly dependent not only on the number of cardiometabolic abnormalities needed to define the metabolically “healthy” status but also on which abnormalities are included (and their cutoffs) (1, 3, 5, 6) and how obesity is defined. This highlights the need to reach a consensus for a universal definition to make comparable findings across different cohorts. We decided to use the most commonly reported list of cardiac abnormalities with their relevant cutoffs (3, 14), but applying more stringent criteria to define the metabolically healthy status. The underlying reasoning was that even though these criteria were historically based on the components of the metabolic syndrome with the addition of an insulin resistance index and serum inflammatory markers (1), we felt that patients with only one cardiometabolic abnormality (ie, diabetes or dyslipidemia) would be misclassified as metabolically healthy. Hence, in this cohort, healthy status describes the absence of any metabolic disorder.

### Comparison with previous studies

The distribution of subclinical atherosclerosis across body size phenotypes is quite linear for CACS, consistent with previous cross-sectional studies reporting computed tomography outcomes in healthy Korean populations (6, 13). In contrast, little difference was observed between metabolically unhealthy overweight and obese subjects in terms of the number of plaques observed by 2DVUS. Although the most plausible explanation is that the increase in BMI has no additive effect to the presence of cardiometabolic abnormalities, it cannot be ruled out a certain degree of misclassification, given that the assessment of subclinical atherosclerosis by 2DVUS is technically harder in obese subjects, particularly in deep territories like the abdominal aorta and iliac arteries.

In this study performed in an asymptomatic cohort of individuals free of overt cardiovascular disease, the presence of subclinical atherosclerosis observed in obese and overweight individuals can be mostly attributed to the coexistence of other cardiovascular risk factors, such as hypertension, diabetes, and dyslipidemia; hence, this suggests that the impact of BMI increments is driven by the cardiometabolic status rather than by the weight itself. This was illustrated by the lack of substantial differences across subjects with the same metabolic health but different BMI. Differences in subclinical atherosclerosis were concentrated between groups divided by metabolic health, suggesting a major role for metabolic health in subclinical atherosclerosis in comparison with weight measures. Currently, there is an intense debate about whether metabolically healthy obesity is a harmless condition. Some reports have shown that metabolically healthy obese subjects are not at increased risk of developing cardiovascular disease over mid-term follow-up periods compared with healthy nonobese (5, 22), although studies with longer follow-up (>10 years) have shown a significantly increased risk of mortality (4). Nevertheless, our findings suggest that BMI might be a suboptimal parameter to evaluate the association between obesity and subclinical atherosclerosis.

### Unanswered questions and future research

There are several open questions related to the natural course of body size phenotypes given the lack of prospective studies addressing and capturing body size phenotypes and transitional statuses (1). It is unclear whether healthy overweight or obese individuals can maintain insulin sensitivity during their entire life or whether these statuses simply represent the delayed onset of obesity-related insulin resistance. It is also unknown what role might play heritable traits or other causal factors leading to transitions from healthy to unhealthy status, and vice versa. Nevertheless, the importance of identifying body size phenotypes relies on the fact that environmental and behavioral factors can modify the healthy and unhealthy phenotypes, and transitions from one to the other phenotype are possible. To date, recommendations for obesity treatment do not consider differences between healthy and unhealthy overweight or obese phenotypes. However, the stratification of obese individuals based on their cardiometabolic phenotype may be important to identify those who are to be prioritized for early pharmacological treatment in addition to lifestyle intervention. There is already some evidence supporting the effect of modifier factors on body size phenotypes. Different calorie-restricted weight loss diets, including low-fat, Mediterranean, and low-carbohydrate diets have been shown to significantly improve metabolic parameters in obese individuals despite only modest weight loss after a 2-year intervention, supporting the hypothesis that caloric restriction may reverse unhealthy into healthy obesity even without large amounts of weight loss (23). Also, it has been shown that bariatric surgery in morbidly obese patients results in long-term weight loss and improvement and/or remission of metabolic diseases and comorbidities, suggesting that the unhealthy obese phenotype can be reversed at least in selected patients (24). Increased physical activity and cardio-respiratory fitness may also contribute to making a healthy transition (25). In this line, a small trial has shown that lifestyle interventions that affect weight loss improve insulin sensitivity and other cardiometabolic risk factors in frail obese older adults. However, continued improvement in insulin sensitivity might only be achieved when exercise training is added to weight loss (26, 27). Further longitudinal studies are needed to evaluate the impact of lifestyle and treatment modifiers on the delay or acceleration of subclinical atherosclerosis.

### Limitations

Associations between body size phenotypes and subclinical atherosclerosis cannot be interpreted as causal relationships given the cross-sectional nature of our data. Despite our efforts to adjust for known risk factors in this cohort through the use of multivariate modeling (12, 28), there may have been residual confounding by unmeasured and measured variables. The PESA study cohort is a relatively homogeneous occupational cohort that may not be representative of the general population. Some limitations have been described for BMI as a measure for obesity—ie, it cannot distinguish between fat and lean tissue. Collection of real fat distribution and volume using imaging techniques would provide more information than BMI and WC. Image quality acquisition by 2DVUS might affect the outcome in obese patients, particularly in deep territories given their suboptimal window. Furthermore, statins, which are part of the criteria defining the healthy status, may impact the outcomes (fewer plaques and more CACS) (29). Importantly, no information was captured regarding the duration of exposure to cardiometabolic abnormalities and BMI categories. We also lacked information on the potential role of adipokines and their distribution across body size phenotype, although it has been described elsewhere that they might have a prognostic value in subjects with and without cardiovascular disease (30).

## Conclusions

Obesity is an expanding worldwide public health epidemic with enormous medical and socioeconomic consequences. Using a thorough approach through several imaging techniques, we have observed an increasing prevalence of subclinical atherosclerosis across BMI categories for metabolically healthy subjects and a steady prevalence in overweight and obese metabolically unhealthy subjects compared to their normal-weight counterparts. When BMI was used to define body size phenotypes, the association of weight with subclinical atherosclerosis appeared to be mostly driven by the coexistence of cardiometabolic risk factors.

## Data Availability

The datasets generated during and/or analyzed during the current study are not publicly available but are available from the corresponding author on reasonable request.

## Abbreviations

2DVUS: two-dimensional vascular ultrasound
BMI: body mass index
CACS: coronary artery calcium score
CES-D: Center for Epidemiological Studies Depression
CCT: cardiac computed tomography
HDL-C: high-density lipoprotein–cholesterol
HOMA-IR: homeostatic model assessment for insulin resistance
hsCRP: high-sensitivity C-reactive protein
IQR: interquartile range
LDL-C: low-density lipoprotein cholesterol
PESA: Progression of Early Subclinical Atherosclerosis
PSS: Perceived Stress Scale
WC: waist circumference

## Additional Information

***Disclosure Summary:*** Dr. Bueno reports grants from Instituto de Salud Carlos III, personal fees from Bayer, personal fees from Novartis, grants, personal fees and nonfinancial support from AstraZeneca, grants and personal fees from BMS-Pfizer, personal fees from MEDSCAPE-the Heart-org, and personal fees from Amgen, outside the submitted work. Dr. Ordovás reports grants from USDA, grants from Archer Daniels Midland, outside the submitted work; and Scientific Advisory Board/consultant for Nutrigenomix, the Predict Study, GNC, and Weight Watchers. The rest of the authors declare that they have no conflict of interests with the present work.

## References

[1] Blüher M . The distinction of metabolically ‘healthy’ from ‘unhealthy’ obese individuals. Curr Opin Lipidol. 2010;21(1):38-43. 19915462 10.1097/MOL.0b013e3283346ccc

[2] Olshansky SJ, Passaro DJ, Hershow RC, et al. A potential decline in life expectancy in the United States in the 21^st^ century. N Engl J Med. 2005;352(11):1138-1145. 15784668 10.1056/NEJMsr043743

[3] Wildman RP, Muntner P, Reynolds K, et al. The obese without cardiometabolic risk factor clustering and the normal weight with cardiometabolic risk factor clustering: prevalence and correlates of 2 phenotypes among the US population (NHANES 1999-2004). Arch Intern Med. 2008;168(15):1617-1624. 18695075 10.1001/archinte.168.15.1617

[4] Kramer CK, Zinman B, Retnakaran R. Are metabolically healthy overweight and obesity benign conditions?: A systematic review and meta-analysis. Ann Intern Med. 2013;159(11):758-769. 24297192 10.7326/0003-4819-159-11-201312030-00008

[5] Rhee EJ, Seo MH, Kim JD, et al. Metabolic health is more closely associated with coronary artery calcification than obesity. PLoS One. 2013;8(9):e74564. 24040286 10.1371/journal.pone.0074564 PMC3770589

[6] Chang Y, Kim BK, Yun KE, et al. Metabolically-healthy obesity and coronary artery calcification. J Am Coll Cardiol. 2014;63(24):2679-2686. 24794119 10.1016/j.jacc.2014.03.042

[7] Guo F, Garvey WT. Cardiometabolic disease risk in metabolically healthy and unhealthy obesity: Stability of metabolic health status in adults. Obesity (Silver Spring). 2016;24(2):516-525. 26719125 10.1002/oby.21344 PMC4731253

[8] Fernández-Ortiz A, Jiménez-Borreguero LJ, Peñalvo JL, et al. The Progression and Early detection of Subclinical Atherosclerosis (PESA) study: rationale and design. Am Heart J. 2013;166(6):990-998. 24268213 10.1016/j.ahj.2013.08.024

[9] Fernández-Friera L, Fuster V, López-Melgar B, et al. Normal LDL-cholesterol levels are associated with subclinical atherosclerosis in the absence of risk factors. J Am Coll Cardiol. 2017;70(24):2979-2991. 29241485 10.1016/j.jacc.2017.10.024

[10] Fernández-Friera L, Peñalvo JL, Fernández-Ortiz A, et al. Prevalence, vascular distribution, and multiterritorial extent of subclinical atherosclerosis in a middle-aged cohort: the PESA (Progression of Early Subclinical Atherosclerosis) Study. Circulation. 2015;131(24):2104-2113. 25882487 10.1161/CIRCULATIONAHA.114.014310

[11] López-Melgar B, Fernández-Friera L, Oliva B, et al. Subclinical atherosclerosis burden by 3D ultrasound in mid-life: the PESA Study. J Am Coll Cardiol. 2017;70(3):301-313. 28705310 10.1016/j.jacc.2017.05.033

[12] Domínguez F, Fuster V, Fernández-Alvira JM, et al. Association of sleep duration and quality with subclinical atherosclerosis. J Am Coll Cardiol. 2019;73(2):134-144. 30654884 10.1016/j.jacc.2018.10.060

[13] Jung CH, Lee MJ, Hwang JY, et al. Association of metabolically healthy obesity (MHO) with subclinical coronary atherosclerosis in a Korean population. Obesity 2014;22(12): 2613-2620. 25155902 10.1002/oby.20883

[14] Gutiérrez-Repiso C, Soriguer F, Rojo-Martínez G, et al. Variable patterns of obesity and cardiometabolic phenotypes and their association with lifestyle factors in the Di@bet.es study. Nutr Metab Cardiovasc Dis. 2014;24(9):947-955. 24984822 10.1016/j.numecd.2014.04.019

[15] Pearson TA, Mensah GA, Alexander RW, et al.; Centers for Disease Control and Prevention; American Heart Association. Markers of inflammation and cardiovascular disease: application to clinical and public health practice: a statement for healthcare professionals from the Centers for Disease Control and Prevention and the American Heart Association. Circulation. 2003;107(3):499-511. 12551878 10.1161/01.cir.0000052939.59093.45

[16] Miller A, Green M, Robinson D. Simple rule for calculating normal erythrocyte sedimentation rate. Br Med J (Clin Res Ed). 1983;286(6361):266. 10.1136/bmj.286.6361.266 PMC1546487 6402065

[17] Touboul PJ, Hennerici MG, Meairs S, et al.; Advisory Board of the 3^rd^ Watching the Risk Symposium 2004, 13^th^ European Stroke Conference. Mannheim intima-media thickness consensus. Cerebrovasc Dis. 2004;18(4):346-349. 15523176 10.1159/000081812

[18] Agatston AS, Janowitz WR, Hildner FJ, Zusmer NR, Viamonte M Jr, Detrano R. Quantification of coronary artery calcium using ultrafast computed tomography. J Am Coll Cardiol. 1990;15(4):827-832. 2407762 10.1016/0735-1097(90)90282-t

[19] Rossello X, Dorresteijn JA, Janssen A, et al. Risk prediction tools in cardiovascular disease prevention: a report from the ESC Prevention of CVD Programme led by the European Association of Preventive Cardiology (EAPC) in collaboration with the Acute Cardiovascular Care Association (ACCA) and the Association of Cardiovascular Nursing and Allied Professions (ACNAP). Eur J Prev Cardiol. 2019;26(14):1534-1544. 31234648 10.1177/2047487319846715

[20] Soler J, Pérez-Sola V, Puigdemont D, Pérez-Blanco J, Figueres M, Alvarez E. [Validation study of the Center for Epidemiological Studies-Depression of a Spanish population of patients with affective disorders]. Actas Luso Esp Neurol Psiquiatr Cienc Afines. 1997;25(4):243-249. 9412163

[21] Remor E . Psychometric properties of a European Spanish version of the Perceived Stress Scale (PSS). Span J Psychol. 2006;9(1):86-93. 16673626 10.1017/s1138741600006004

[22] Hamer M, Stamatakis E. Metabolically healthy obesity and risk of all-cause and cardiovascular disease mortality. J Clin Endocrinol Metab. 2012;97(7):2482-2488. 22508708 10.1210/jc.2011-3475 PMC3387408

[23] Shai I, Schwarzfuchs D, Henkin Y, et al.; Dietary Intervention Randomized Controlled Trial (DIRECT) Group. Weight loss with a low-carbohydrate, Mediterranean, or low-fat diet. N Engl J Med. 2008;359(3):229-241. 18635428 10.1056/NEJMoa0708681

[24] Sjöström L, Narbro K, Sjöström CD, et al.; Swedish Obese Subjects Study. Effects of bariatric surgery on mortality in Swedish obese subjects. N Engl J Med. 2007;357(8): 741-752. 17715408 10.1056/NEJMoa066254

[25] Ortega FB, Lee DC, Katzmarzyk PT, et al. The intriguing metabolically healthy but obese phenotype: cardiovascular prognosis and role of fitness. Eur Heart J. 2013;34(5):389-397. 22947612 10.1093/eurheartj/ehs174 PMC3561613

[26] Bouchonville M, Armamento-Villareal R, Shah K, et al. Weight loss, exercise or both and cardiometabolic risk factors in obese older adults: results of a randomized controlled trial. Int J Obes (Lond). 2014;38(3):423-431. 23823329 10.1038/ijo.2013.122 PMC3835728

[27] Armamento-Villareal R, Aguirre L, Napoli N, et al. Changes in thigh muscle volume predict bone mineral density response to lifestyle therapy in frail, obese older adults. Osteoporos Int. 2014;25(2):551-558. 23892583 10.1007/s00198-013-2450-2 PMC3903658

[28] Peñalvo JL, Fernández-Friera L, López-Melgar B, et al. Association between a social-business eating pattern and early asymptomatic atherosclerosis. J Am Coll Cardiol. 2016;68(8):805-814. 27539172 10.1016/j.jacc.2016.05.080

[29] Ahmadi A, Argulian E, Leipsic J, Newby DE, Narula J. From subclinical atherosclerosis to plaque progression and acute coronary events: JACC state-of-the-art review. J Am Coll Cardiol. 2019;74(12):1608-1617. 31537271 10.1016/j.jacc.2019.08.012

[30] Sans-Roselló J, Casals G, Rossello X, et al. Prognostic value of plasma apelin concentrations at admission in patients with ST-segment elevation acute myocardial infarction. Clin Biochem. 2017;50(6):279-284. 27889567 10.1016/j.clinbiochem.2016.11.018

